# Trend in physical activity patterns of pregnant women living in Brazilian capitals

**DOI:** 10.11606/s1518-8787.2022056003300

**Published:** 2022-05-18

**Authors:** Ana Elisa Madalena Rinaldi, Janaína Aparecida de Paula, Maiara Aparecida Miálich Almeida, José Eduardo Corrente, Maria Antonieta Barros Leite Carvalhaes

**Affiliations:** I Universidade Federal de Uberlândia Faculdade de Medicina Programa de Pós-Graduação em Ciências da Saúde Uberlândia MG Brasil Universidade Federal de Uberlândia. Faculdade de Medicina. Programa de Pós-Graduação em Ciências da Saúde. Uberlândia, MG, Brasil; II Universidade Estadual Paulista Faculdade de Medicina de Botucatu Departamento de Enfermagem Botucatu SP Brasil Universidade Estadual Paulista. Faculdade de Medicina de Botucatu. Departamento de Enfermagem. Programa de Pós-Graduação em Enfermagem. Botucatu, SP, Brasil; III Universidade Estadual Paulista Faculdade de Medicina de Botucatu Escritório de Apoio à Pesquisa Botucatu SP Brasil Universidade Estadual Paulista. Faculdade de Medicina de Botucatu. Escritório de Apoio à Pesquisa. Botucatu, SP, Brasil

**Keywords:** Pregnant Women, Exercise, Leisure Activities, Socioeconomic Factors, Health Surveys

## Abstract

**OBJECTIVES:**

To describe the trend of physical activity (PhA) in four domains performed by pregnant women living in Brazilian capitals and the Federal District (FD) and to verify the association between sociodemographic factors and the practice of leisure-time physical activity between 2007 and 2017.

**METHODS:**

Time trend study carried out with data from the Surveillance System of Risk Factors for chronic diseases by telephone survey (Vigitel). A total of 3,730 pregnant women were interviewed in the period, considering sociodemographic variables (age, macro-region, work, marital status, schooling, skin color) and physical activity in the four domains (leisure-time, work, commuting, domestic – yes/no). For pregnant women who performed leisure-time physical activity, its duration was inquired, expressed in the variable PhA ≥ 150 minutes/week (yes/no). The time trend was evaluated by variance-weighted linear regression (average annual variation was expressed in percentage points – pp) and the association of sociodemographic factors with leisure-time physical activity by Poisson regression, with prevalence ratio (PR) estimation.

**RESULTS:**

The prevalence of pregnant women with 12 years of schooling or more increased in the analyzed period (+1.37 pp/year), as well as the prevalence of pregnant women with more than 35 years of age (+1.11 pp/year) and those who work (+0.75 pp/year). The prevalence of leisure-time physical activity by pregnant women increased from 29.3% in 2007 to 37.6% in 2017 (+1.37 pp/year), and of PhA ≥ 150min/week from 2.3% to 20.6% (+2.33 pp/year), respectively, and there was a reduction in domestic physical activities from 63.9% to 38.9% (-1.65 pp/year). The prevalence of leisure-time physical activity was higher among pregnant women with more than 12 years of schooling (PR = 2.22; 95%CI: 1.73–2.84) as compared to those with less than 8 years of age, and lower among black/brown/indigenous pregnant women, compared to white/yellow ones (PR = 0.87; 95%CI: 0.78–0.97). The prevalence of PhA ≥ 150min/week increased according to years of schooling and age.

**CONCLUSIONS:**

The prevalence of leisure-time physical activity and its performance for ≥ 150 minutes/week increased in the analyzed period, and both were directly associated with greater schooling.

## INTRODUCTION

The current recommendation for the practice of at least 150 minutes of moderate intensity physical activity (PhA) per week from the World Health Organization (WHO)^1^, endorsed by the Brazilian Ministry of Health, has also been shown to be beneficial for pregnant women, as long as there are no contraindications^2^. It is recommended that these 150 minutes be distributed throughout the week during the three trimesters of pregnancy^2 , 3^.

The benefits resulting from the practice of physical activity involve protection against such adverse obstetric and neonatal outcomes as excessive gestational weight gain^4^, arterial hypertension^5^, gestational diabetes mellitus^4 , 6^, surgical delivery^5^, and macrosomia^4^. The types of PhA during pregnancy most frequently studied in the literature, considered safe and beneficial, include aerobic exercises, resistance and stretching exercises, cycling on a cycle ergometer, swimming and water aerobics, dance, and pilates^7^.

The relationship between occupational PhA of the pregnant woman and maternal-fetal health outcomes still need to be investigated, due to the divergent and limited results of published studies^2^. The effects of occupational PhA during pregnancy on health outcomes seem to be dependent on the weight load handled throughout the day, and lifting weights greater than 20 kg more than 10 times a day presented an increased risk of preterm birth. The recommendation of the National Institute for Occupational Safety and Health can be used to estimate the maximum acceptable weight limit for a pregnant woman, considering occupational activity and gestational age^8 , 9^.

The percentage of pregnant women who regularly practice leisure-time physical activity is low, according to Brazilian regional studies carried out in the 2000s and 2010s^10^. In Pelotas (RS) (2004), 12.9% of pregnant women reported practicing some type of PhA and there was a reduction in their practice in the last trimester^11^. In the interior of São Paulo, the percentage of pregnant women interviewed in 2010 and in the period 2011–2014 who claimed to have moderate PhA ranged from 12.5%^10^to 7.6%^12^, respectively. With this picture, it is noted that in the Low-Risk Prenatal Care Manual recommendations from the Ministry of Health (2012)^13^, in force to date, there is little emphasis on the promotion of physical activity during pregnancy and no emphasis on its potential as a protective behavior against obstetric and neonatal complications^13^.

There are no representative data on the Brazilian population of pregnant women and, consequently, the temporal trend of PhA practice during pregnancy is unknown, although monitoring the physical activity of the population in general is one of the priority actions of the National Health Promotion Policy^14^. A Brazilian study was identified that compared two groups (2004 and 2015) of pregnant women in Pelotas (RS). There was an increase in the percentage of active pregnant women before becoming pregnant, stability of the percentage of active pregnant women in the first two gestational trimesters, and a reduction in the active proportion in the third trimester^15^.

The Surveillance System for Risk Factors for chronic diseases by telephone survey (Vigitel) provides data on physical activity. The presence of pregnant women in the annual samples of Vigitel is an opportunity to get to know the panorama of PhA practice in all Brazilian regions, even if restricted to those who live in the capitals and the Federal District (FD). Thus, our objectives were to describe the time trend of PhA in pregnant women living in Brazilian capitals and in the FD, between 2007 and 2017, and to verify the association between sociodemographic factors and the scope of the recommendation of leisure-time PhA by pregnant women based on Vigitel data.

## METHODS

### Study Design, Data Source and Sample

This is a time trend study with annual data from Vigitel, from 2007 to 2017, with each survey having a cross-sectional design. Vigitel’s objective is to continuously and frequently monitor the distribution of risk and protective factors for chronic non-communicable diseases (NCDs).

The Vigitel Project was approved by the National Research Ethics Commission (Conep) for Human Beings of the Ministry of Health (Opinion 355590, CAAE: 16202813.2.0000.0008) and the databases are public and available on the Ministry of Health website.

The annual sample is obtained by drawing lots of five thousand telephone lines per city, systematically and stratified by zip code. The lines then undergo a second draw and are divided into replicas of 200 lines, excluding lines corresponding to companies, those that are out of service or that do not respond to six call attempts made on different days and times. The eligible ones go through a second stage, with a drawing of an adult resident to answer the questionnaire^16^. The adoption of the rake method to obtain the post-stratification weight allows inference of estimates for the adult population residing in Brazilian capitals and FD, regardless of whether the household has a telephone line or not. This method was initially adopted in 2012, but was calculated and made available from 2006 onwards^17^.

A total of 3,730 women reported being pregnant between 2007 and 2017. Due to the sample being composed of pregnant women, a new rake weight was constructed from the variable “internal weight”, already available in the databases (this variable is constructed by multiplying the inverse of the probability of adults in the household by the inverse of the probability of telephone lines in the household), the age group of pregnant women (< 20 years; 20–34.9 years and ≥ 35 years) and the capital where they lived. A variable was constructed corresponding to the number of pregnant women by age group and by capital. Subsequently, the rake weight was generated from these three variables. The need for a new weight is due to the small number of pregnant women in the sample of each survey in the analyzed period and also due to the drop in the fertility rate (from two points in 2006 to approximately 1.75 in 2015)^18^and birth rate (15.8 births/thousand inhabitants in 2006 to 13.8 births/thousand inhabitants in 2016)^19^.

### Sociodemographic Variables

The sociodemographic variables selected were: age (< 20 years, 20 to 34.9 years, ≥ 35 years); schooling (zero to eight years, nine to 11 years, ≥ 12 years); work (yes/no); marital status (single, widowed/divorced, and married/informally married), macro-region (North, Northeast, Midwest, Southeast and South) and skin color (white, yellow, black, brown or indigenous). In the analyses, we chose to group the pregnant women into two categories, one for women who self-declared as white and yellow and the other for those who self-declared as black, brown and indigenous^20^.

### Physical Activity Variables

The variables available in the Vigitel databases and analyzed in this study refer to physical activity in four domains: occupational, domestic, commuting and leisure-time. For the occupational, domestic and commuting PhA domains, we used the indicator variables previously available in all databases (2007 to 2017) and whose configuration was “no/yes”.

The “yes” category for occupational PhA corresponds to the pregnant woman having worked in the last three months and having reported walking a lot and/or carrying weight at work. The “yes” category for PhA when commuting corresponds to the pregnant woman having walked 30 minutes or more between work and/or course/school. The “yes” category for domestic PhA corresponds to performing the cleaning alone and/or doing the heavy part of the cleaning alone.

PhA in leisure-time was configured by the authors from the variable “In the last three months, did you practice any kind of physical exercise or sport?” (yes/no). The variable type of leisure-time PhA was only available for pregnant women who answered that they had practiced some type of exercise or sport in the last three months and had done it more than once a week. The different PhA modalities were grouped into four categories, according to the classification of energy expenditure intensity in multiples of metabolic equivalents (MET)^21^: walking on the street and on the treadmill; dance, gymnastics, water aerobics, weight training, swimming; bicycle, soccer, martial arts, volleyball, tennis; street and treadmill running.

The leisure-time PhA variable ≥ 150min per week (PhA ≥ 150min/week) was configured by the authors from the combination of two variables: weekly PhA frequency (number of days/week) and PhA duration (minutes/section). Pregnant women who reported practicing some exercise/sport in the last three months more frequently than once a week and whose sum of weekly frequency and duration resulted in ≥ 150min/week were included in the “yes” category. Pregnant women who did not do physical activity or who did it only once a week were classified as “no” for this variable. Thus, it was possible to estimate the prevalence of physical activity as recommended by the WHO^1^.

### Data Analysis

Data were described in terms of prevalence by year of the survey and the temporal trend analysis was performed using variance-weighted linear regression. The average rate of increase or decrease (expressed in percentage points – pp) of prevalence between 2007 and 2017 corresponds to the value of the slope coefficient of the regression with the respective p-value of trend (significance level less than 5%).

The prevalence of leisure-time physical activity (yes/no) and of PhA practice ≥ 150min/week (yes/no), according to schooling, were illustrated in a graph defined as equiplot.

The association between the pregnant woman’s level of education and the performance of leisure-time physical activity and PhA ≥ 150min/week was estimated by Poisson regression analysis, adjusted for age group, color, geographic macro-region and marital status. Two models were adjusted for each outcome: a) model 1 with the inclusion of all survey years (2007 to 2017) and b) model 2 with sample division into three periods (2007 to 2009; 2010 to 2014 and 2015 to 2017). The option to analyze the models in these three periods is due to the reduction of the sample over the years, leading to an increase in the confidence intervals in the analyses and a reduction in the precision of the estimates. All analyses were performed using the Stata SE 13.0^®^ program.

## RESULTS

The total sample collected from 2007 to 2017 comprised 3,730 pregnant women (variation from 2% to 1.38% of the total sample of women interviewed in each survey), with similar and larger sizes in the 2007 period (n = 462 pregnant women) to 2011 (n = 396 pregnant women) and a reduction from 2012 (n = 317 in 2012 to n = 188 in 2017).

We observed an increase in the percentage of pregnant women aged ≥ 35 years in the analyzed period, but the majority of pregnant women were between 20 and 34.9 years old during the period. There was a significant increase in the prevalence of pregnant women with ≥ 12 years of schooling, with the majority in 2017 being classified in this schooling range, in contrast to 2007, when the category of nine to 11 years of schooling was more prevalent. The prevalence of pregnant women who reported being working increased from 58.6% to 67.8% between 2007 and 2017. The highest prevalence of pregnant women in the period from 2007 to 2017 was identified in the North and Northeast regions, with an increase, in the same period, of those living in the Northeast region and a reduction in the Midwest and South. Most pregnant women reported being married or in a common-law marriage. There was a higher prevalence, throughout the analyzed period, of pregnant women who self-declared as black, mixed race and indigenous (59% in 2007 and 61.3% in 2017) ( Table 1 ).

**Table 1:** Sociodemographic characteristics of Brazilian pregnant women living in Brazilian capitals and the Federal District, according to year of the survey. Vigitel, 2007–2017.

Variables	Survey years											
	2007	2008	2009	2010	2011	2012	2013	2014	2015	2016	2017	Average increment/year (in pp) (p)
Age range (years)												
< 20	5.7	4.6	4.9	7.5	8.8	5.9	5.4	3.9	2.6	7.2	6.2	-0.12 (0.288)
20 to 35	74.7	73.7	76.3	71.9	75.4	74.4	69.4	68.7	69.1	65.7	59.4	-1.09 (< 0.001)
≥ 35	19.6	21.7	18.8	20.6	15.8	19.7	25.2	27.4	28.3	27.1	34.4	1.11 (< 0.001)
Schooling (years)												
0 to 8	17.2	14.9	11.3	18.4	17.3	12.6	11.6	13.1	8.9	7.5	8.9	-0.79 (< 0.001)
9 to 11	49.0	46.2	50.9	47.8	46.7	48.7	46.6	45.7	49.7	39.0	40.8	-0.59 (0.040)
≥ 12	33.8	38.9	37.8	33.8	36.0	38.7	41.8	41.2	41.4	53.5	50.3	1.37 (< 0.001)
Work												0.75 (0.007)
No	41.4	36.6	37.1	37.2	40.7	38.8	39.0	37.6	27.1	35.0	32.2	
Yes	58.6	63.4	62.9	62.8	59.3	61.2	61.0	62.4	72.9	65.0	67.8	
Macro-region												
North	31.7	31.3	38.4	36.0	34.4	31.5	34.8	27.9	30.8	31.4	31.6	-0.29 (0.295)
Northeast	26.3	33.6	26.9	26.6	32.0	33.0	29.4	36.6	40.9	30.2	39.3	0.97 (< 0.001)
Midwest	19.2	13.0	15.0	15.2	16.3	13.5	13.4	13.4	10.3	15.8	11.0	-0.48 (0.015)
Southeast	12.9	14.2	11.8	11.7	10.2	12.4	12.0	14.9	12.1	14.5	14.3	0.08 (0.705)
South	9.9	7.9	7.9	10.5	7.1	9.6	10.4	7.2	5.9	8.1	3.8	-0.41 (0.003)
Marital status												0.49 (0.075)
Single, widow, divorced	23.5	33.9	32.6	31.7	35.7	28.3	34.5	28.8	27.9	37.2	34.9	
Married, common-law marriage	76.5	66.1	67.4	68.3	64.3	71.7	65.5	71.2	72.1	62.8	65.1	
Skin color												-0.08 (0.782)
White/yellow	41.0	35.7	41.7	39.4	40.8	44.7	40.6	39.4	36.6	43.5	38.7	
Black, brown, indigenous	59.0	64.3	58.3	60.6	59.2	55.3	59.4	60.6	63.4	56.5	61.3	

pp: percentage points.

The prevalence of pregnant women who reported practicing some physical activity at work (occupational PhA) was stable over the period, around 32%. There was a slight increase in PhA when commuting between home and work/study over the period (+0.34 pp/year) and a significant reduction in physical activity performed in domestic work (-1.65 pp/year). We observed an increase in PhA classified as leisure-time (+1.37 pp/year), as well as in the percentage of pregnant women who reported practicing PhA ≥ 150min/week (+2.33 pp/year) ( Table 2 ). We found that, in 2007, domestic PhA was the most prevalent, with data similar to leisure-time and occupational PhA. However, in 2017, the prevalence of domestic PhA and leisure-time PhA was virtually equal to and higher than occupational and commuting PhA.

**Table 2:** Trend in PhA practice in the four domains (leisure-time, work, commuting and domestic) and PhA practice according to the WHO recommendation (150 min/week) of Brazilian pregnant women living in Brazilian capitals and the Federal District, according to year of the survey. Vigitel, 2007–2017.

Variables	Survey years											
	2007	2008	2009	2010	2011	2012	2013	2014	2015	2016	2017	Average increment/year (in pp) (p)
Work												0.15 (0.593)
No	68.3	62.9	65.7	62.3	65.5	64.7	66.6	64.2	59.3	64.3	68.4	
Yes	31.7	37.1	34.3	37.7	34.5	35.3	33.4	35.8	40.7	35.7	31.6	
Commuting												0.34 (0.038)
No	94.9	89.3	88.6	84.2	85.8	88.7	89.6	88.1	90.1	90.7	91.3	
Yes	5.1	10.7	11.4	15.8	14.2	11.3	10.4	11.9	9.9	9.3	8.7	
Domestic activities												-1.65 (< 0.001)
No	36.1	52.5	48.9	48.3	49.6	51.2	54.2	51.3	56.9	55.4	61.6	
Yes	63.9	47.5	51.1	51.7	50.4	48.8	45.8	48.7	43.1	44.6	38.4	
Leisure-time												1.37 (< 0.001)
No	70.7	75.8	71.4	76.9	77.6	69.5	66.0	66.8	64.5	56.6	62.4	
Yes	29.3	24.2	28.6	23.1	22.4	30.5	34.0	33.2	35.5	43.4	37.6	
PhA (> 150 min/week)												2.33 (< 0.001)
No	97.7	87.1	84.2	87.7	87.3	82.5	78.8	82.1	78.3	73.7	79.4	
Yes	2.3	12.9	15.8	12.3	12.7	17.5	21.2	17.9	21.7	26.3	20.6	

PhA: physical activity; pp: percentage points.

As the types of leisure-time PhA practiced by pregnant women were similar in all years, we chose to describe the results of the entire period. The most prevalent activities were walking on the street and on the treadmill (57.8%); followed by the category “dance, gymnastics, water aerobics, weight training and swimming” (32.7%); with lower prevalence of the categories “bicycle, soccer, martial arts, tennis and volleyball” (7.2%) and “running on the street and on the treadmill” (2.3%).

The prevalence of leisure-time PhA and PhA ≥ 150min/week were higher among pregnant women with ≥ 12 years of schooling in the analyzed period. For these two variables, the prevalence was higher than 30% among pregnant women with higher education. Among pregnant women with less than eight years of schooling, the prevalence of PhA ≥ 150min/week was less than 10% in almost all years of the analyzed period ( Figure ).

**Figure:**
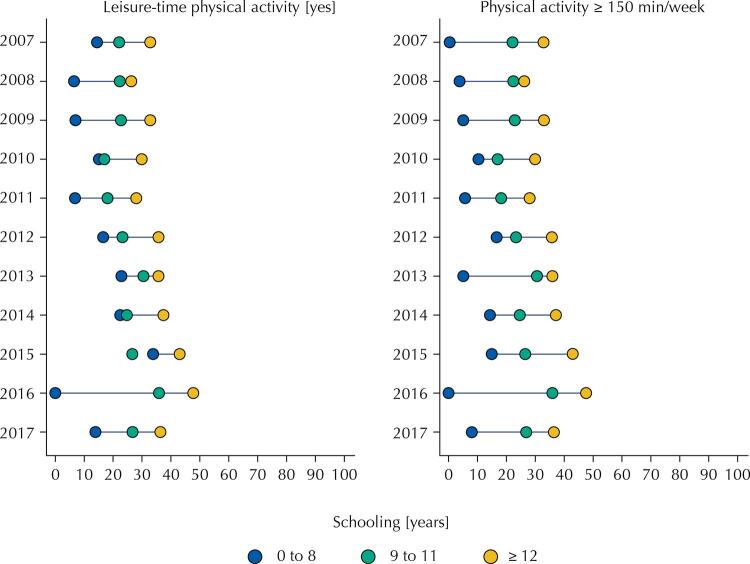
Equiplot of leisure-time physical activity (PhA) and PhA practice for 150 min/week or more according to schooling in pregnant women residing in Brazilian capitals and the Federal District. Vigitel, 2007–2017.

There was an increase in the practice of leisure-time physical activities and PhA ≥ 150min/week from 2007 to 2017 ( Table 3 ). We found that leisure-time PhA practice was 2.22 times more prevalent among pregnant women with ≥ 12 years of schooling compared to those with less than eight years (model 1). This pattern was repeated in the three analyzed periods (model 2). Pregnant women who self-declared themselves as black/brown/indigenous reported a lower prevalence of physical activity in model 1 and in the period from 2015 to 2017 (model 2). We did not observe a difference in prevalence according to geographic region and age (models 1 and 2).

**Table 3:** Association between schooling and leisure-time PhA practice in the last 3 months and PhA for 150min/week or more in pregnant women living in Brazilian capitals and the Federal District, in a period of 10 years (model 1) and annual intervals (model 2 ). Vigitel, 2007–2017.

	PhA at leisure-time				PhA > 150min/week			
	Model 1	Model 2			Model 1	Model 2		
	2007–2017 PR (95%CI)	2007–2009 PR (95%CI)	2010–2014 PR (95%CI)	2015–2017 PR (95%CI)	2007–2017 PR (95%CI)	2007–2009 PR (95%CI)	2010–2014 PR (95%CI)	2015–2017 PR (95%CI)
Year	1.04 (1.02–1.06)				1.11 (1.08–1.14)			
Schooling (years)								
0 to 8	1.00 (ref)	1.00 (ref)	1.00 (ref)	1.00 (ref)	1.00 (ref)	1.00 (ref)	1.00 (ref)	1.00 (ref)
9 to 11	1.66 (1.30–2.13)	1.96 (1.30–2.95)	1.53 (1.09–2.16)	1.60 (0.79–3.22)	1.93 (1.33–2.80)	3.98 (1.82–8.73)	1.55 (0.98–2.46)	2.15 (0.86–5.37)
≥ 12	2.22 (1.73–2.84)	2.41 (1.60–3.64)	2.12 (1.51–2.98)	2.29 (1.15–4.58)	2.36 (1.63–3.42)	4.56 (2.08–10.0)	1.95 (1.22–3.09)	2.84 (1.16–6.99)
Skin Color								
White/yellow	1.00 (ref)	1.00 (ref)	1.00 (ref)	1.00 (ref)	1.00 (ref)	1.00 (ref)	1.00 (ref)	1.00 (ref)
Black, brown, indigenous	0.87 (0.78–0.97)	0.86 (0.70–1.05)	1.00 (0.84–1.19)	0.64 (0.52–0.79)	0.91 (0.76–1.08)	0.81 (0.57–1.16)	1.03 (0.80–1.32)	0.79 (0.56–1.07)
Macro-region								
North	1.00 (ref)	1.00 (ref)	1.00 (ref)	1.00 (ref)	1.00 (ref)	1.00 (ref)	1.00 (ref)	1.00 (ref)
Northeast	0.85 (0.74–0.98)	0.82 (0.64–1.04)	0.81 (0.64–1.02)	0.97 (0.75–1.24)	0.86 (0.69–1.08)	0.66 (0.41–1.06)	0.93 (0.66–1.32)	0.97 (0.66–1.42)
Midwest	1.15 (0.98–1.34)	1.02 (0.77–1.34)	1.23 (0.97–1.56)	1.24 (0.93–1.65)	1.34 (1.05–1.70)	0.93 (0.57–1.53)	1.54 (1.08–2.19)	1.52 (0.99–2.32)
Southeast	1.03 (0.86–1.23)	0.86 (0.62–1.20)	1.28 (1.00–1.65)	0.86 (0.61–1.21)	1.18 (0.90–1.53)	0.82 (0.46–1.47)	1.53 (1.05–2.23)	1.08 (0.66–1.75)
South	1.19 (0.99–1.44)	1.19 (0.87–1.61)	1.36 (1.02–1.80)	0.86 (0.57–1.31)	1.43 (1.06–1.92)	1.25 (0.73–2.15)	1.75 (1.14–2.67)	1.01 (0.55–1.85)
Age (years)								
< 20	1.00 (ref)	1.00 (ref)	1.00 (ref)	1.00 (ref)	1.00 (ref)	1.00 (ref)	1.00 (ref)	1.00 (ref)
20 to 34	1.15 (0.88–1.50)	0.95 (0.62–1.47)	1.51 (0.96–2.38)	0.82 (0.51–1.32)	1.78 (1.10–2.88)	1.55 (0.60–3.99)	1.72 (0.88–3.36)	2.15 (0.80–5.82)
≥ 35	1.28 (0.97–1.69)	1.05 (0.66–1.67)	1.83 (1.14–2.94)	0.82 (0.50–1.34)	2.17 (1.32–3.58)	2.16 (0.81–5.78)	2.40 (1.19–4.80)	2.12 (0.77–5.80)

PhA: physical activity; PR: prevalence ratio; 95%CI: 95% confidence interval.

The practice of PhA ≥ 150min/week was 2.36 times more prevalent among pregnant women with ≥ 12 years of schooling compared to those with less than eight years (model 1), with prevalence ratio values being higher, especially in the period from 2007 to 2009. We also observed a higher prevalence of PhA in pregnant women aged > 20 years (model 1) and those residing in the Midwest and South regions (model 1, model 2 – 2010 to 2014) ( Table 3 ).

## DISCUSSION

In this study, we observed a significant reduction in the number of pregnant women who reported doing PhA at home, an increase in those who did PhA in leisure-time time and who reached 150min/week in the analyzed period. However, we also observed that the practice of PhA in leisure-time time, as well as PhA ≥ 150min/week, are higher among pregnant women with greater schooling, indicating important social inequality among Brazilian pregnant women.

Occupational PhA remained stable over the analyzed decade and PhA in commuting showed a small increase. The percentage of pregnant women who reported doing domestic physical activities was reduced by half. The most significant increase in the period was in the leisure-time PhA domain, a component of the PhA set that is more modifiable by health promotion actions.

The relationship between domestic and occupational physical activities and the risk of preterm birth, low birth weight and intrauterine growth restriction dominated research in the 1980s and 1990s, especially in low-income countries, where such outcomes were and still are a public health issue. The outcomes of that time indicated that the excess hours of standing, going up and down stairs and carrying weight on a daily basis reduces intrauterine growth and gestational age at delivery^22^. These outcomes are still accepted and, therefore, the reduction of active pregnant women in these domains can be seen as positive^2^.

It is possible that the effects of the different domains of physical activities vary according to the pregnant women’s life context, as indicated by two recent studies^23 , 24^. In two cohort studies with French pregnant women, high levels of domestic PhA and sedentary activities in the third trimester of pregnancy increased the chances of postpartum depression, while exercise and sports activities were not associated with this outcome^23^. In a Vietnamese cohort, higher levels of domestic and occupational activities were associated with lower gestational weight gain. In the local context, this outcome was considered favorable to the prevention of gestational diabetes, a frequent problem in this population, but negative for the occurrence of low birth weight newborns^24^.

On the other hand, the literature contains solid evidence on the benefits of regular leisure-time PhA and exercise during pregnancy^2^, reinforcing the positive aspects of the observed trends. However, the average increase was higher at the beginning of the period (2007–2009) (4.5 pp/year) and lower at the end (0.55 pp/year) (2015 and 2017).

There is no national or international goal to reach a certain percentage of pregnant women performing leisure-time PhA ≥ 150 minutes/week. It is possible to estimate that the rate of increase observed in the period 2007–2017 is low if we take into account that, if this pattern is maintained, it will take more than 40 years for half of Brazilian pregnant women to reach the recommendation. This time may be even longer if the rate of increase is the one observed in more recent years (2014 to 2017). This picture indicates the size of the challenge to be faced in Brazil to promote and support women to be physically active during pregnancy. This situation highlights the importance of, in addition to monitoring, identifying the determinants of leisure-time physical activity among women during pregnancy, in order to provide evidence for the conduct of interventions.

Barriers to performing leisure-time PhA by pregnant women have been investigated^24 , 25^. A literature review of 26 qualitative and quantitative studies on barriers to physical activity during pregnancy identified, in all of them, intrapersonal factors that prevent the practice, including tiredness and physical discomfort, in addition to excess activities and responsibilities with work and care for the family. Interpersonal factors were also frequent, with emphasis on the lack of knowledge and guidance on the importance and safety of physical activity in this period^26^. These barriers could be removed by professionals responsible for prenatal care. Barriers related to the organizational and political environment and climate (too cold or too hot, rainy) were also identified by studies, especially insecurity on the streets, lack of public spaces for physical activity, and the high cost of gyms^27^.

Thus, to overcome the barriers, actions in health services and intersectoral actions that go beyond the advice provided by health professionals are necessary. Removing such barriers also implies new perspectives for urban planning, work environments, sharing of domestic work, among other spheres of human life that need to become more favorable to the practice of physical activity by all, including pregnant women. Public policies in all these spheres are necessary, mainly, to support self-declared black, brown and indigenous pregnant women and those with less schooling.

The increase in schooling of pregnant women tends to be favorable for reaching the recommendation of PhA during leisure-time, as previous studies have also verified^12 , 28^. The positive association between greater schooling and leisure-time PhA can be explained by the more favorable socioeconomic status, which affords them to work in activities that require less physical effort, to get help with housework and childcare, that is, having more time and willingness to exercise in their leisure-time^31^. In addition, pregnant women with a higher educational level may have more access to technical information on physical activity during pregnancy, have easier access to safer places with better infrastructure for leisure-time PhA practice. Differences in leisure-time PhA between white/yellow and black/brown/indigenous pregnant women point to inequality related to institutional racism, regardless of schooling, in leisure-time PhA practice.

As strengths of our study, we highlight the production of unprecedented data on the profile of physical activity of pregnant women in Brazilian capitals, thus expanding the knowledge of this profile for the five macro-regions. To date, previous studies on physical activity in pregnant women were based on regional data^10 , 11 , 15^. Another strength is the use of data from a monitoring system (Vigitel) available with the same questions in all years of the surveys, which allowed comparability between the years analyzed.

We identified as the main limitation the absence of some specific and relevant variables when carrying out studies with pregnant women, especially gestational age. This variable would allow us to identify differences regarding the practice of physical activity by gestational trimesters and nutritional status. Vigitel does not focus on pregnant women, but on the adult population residing in Brazilian capitals and the Federal District. Even so, we suggest that the gestational age be asked in future surveys, since Vigitel is the only population-based survey currently available in Brazil that allows the analysis of physical activity in pregnant women. This simple information will allow advances in the monitoring of risk and protective behaviors also for pregnant women and the design of specific interventions for each stage of pregnancy.

We can also highlight that the sample of pregnant women in this study is not representative of the population of pregnant women residing in Brazilian capitals, since the population to be represented is individuals aged 18 years or older. One of the possible explanations for the reduction in the sample size is the drop in fertility over this decade, especially in the capitals.

Another limitation to be considered is the self-report of physical activity compared to direct PhA measurements, performed by pedometers and/or accelerometers. However, trend and monitoring studies that use the same information collection methods are comparable. We emphasize that when classifying pregnant women as to the frequency and weekly duration of leisure-time PhA, we considered all modalities as moderate, since a previous study^12^indicates a low frequency of pregnant women who perform activities of vigorous intensity.

In summary, we found a trend towards an increase in physical activity among pregnant women in the domains of leisure-time and commuting and a significant reduction in domestic activities. Additionally, there was an upward trend in those who reached the recommendation of ≥ 150min/week. However, this increase was decreasing in the period and uneven among pregnant women, according to schooling, being higher among pregnant women with 12 years of schooling or more and among those who self-declared as white/yellow throughout the analyzed period.
