# Supplementary material for: Trend in physical activity patterns of pregnant women living in Brazilian capitals
Source: Rev Saude Publica. 2022 May 18;56:42. doi: 10.11606/s1518-8787.2022056003300 (PMC9126577; doi:10.11606/s1518-8787.2022056003300)
Supplement: Translation

# Translation: Tendência dos padrões de atividade física de gestantes residentes nas capitais brasileiras

**Authors:** Ana Elisa Madalena Rinaldi, Janaína Aparecida de Paula, Maiara Aparecida Miálich Almeida, José Eduardo Corrente, Maria Antonieta Barros Leite Carvalhaes

**Affiliations:** I Brasil Universidade Federal de Uberlândia. Faculdade de Medicina. Programa de Pós-Graduação em Ciências da Saúde. Uberlândia, MG, Brasil; II Brasil Universidade Estadual Paulista. Faculdade de Medicina de Botucatu. Departamento de Enfermagem. Programa de Pós-Graduação em Enfermagem. Botucatu, SP, Brasil; III Brasil Universidade Estadual Paulista. Faculdade de Medicina de Botucatu. Escritório de Apoio à Pesquisa. Botucatu, SP, Brasil

**Keywords:** Gestantes, Exercício Físico, Atividades de Lazer, Fatores Socioeconômicos, Inquéritos Epidemiológicos

## Abstract

**OBJETIVOS:**

Descrever a tendência da atividade física (AF) em quatro domínios realizada por gestantes residentes nas capitais brasileiras e no Distrito Federal (DF) e verificar a associação entre fatores sociodemográficos e a prática de atividade física de lazer entre 2007 e 2017.

**MÉTODOS:**

Estudo de tendência temporal realizado com dados do Sistema de Vigilância de Fatores de Risco para doenças crônicas por inquérito telefônico (Vigitel). Foram estudadas 3.730 gestantes no período, considerando as variáveis sociodemográficas (idade, macrorregião, trabalho, estado civil, escolaridade, cor) e de atividade física nos quatro domínios (lazer, trabalho, deslocamento, doméstico – sim/não). Para as gestantes que realizavam atividade física de lazer, foi questionada a duração, expressa na variável AF ≥ 150 minutos/semana (sim/não). A tendência temporal foi avaliada por regressão linear ponderada pela variância (variação anual média foi expressa em pontos percentuais – p.p.) e a associação de fatores sociodemográficos com a atividade física de lazer por regressão de Poisson, com estimativa da razão de prevalência (RP).

**RESULTADOS:**

A prevalência de gestantes com 12 anos ou mais de estudo aumentou no período analisado (+1,37 p.p./ano), assim como a prevalência de gestantes com mais de 35 anos (+1,11 p.p./ano) e aquelas que trabalham (+0,75 p.p./ano). A prevalência de atividade física de lazer pelas gestantes aumentou de 29,3%, em 2007, para 37,6%, em 2017 (+1,37 p.p./ano), e de AF ≥ 150min/semana de 2,3% para 20,6% (+2,33 p.p./ano), respectivamente, e houve redução de atividades físicas domésticas de 63,9% para 38,9% (-1,65 p.p./ano). A prevalência da prática de atividade física de lazer foi superior entre as gestantes com mais de 12 anos de estudo (RP = 2,22; IC95% 1,73–2,84), quando comparadas àquelas com menos de 8 anos, e menor entre gestantes pretas/pardas/indígenas, comparadas às brancas/amarelas (RP = 0,87; IC95% 0,78–0,97). A prevalência da AF ≥ 150min/semana aumentou conforme os anos de escolaridade e idade.

**CONCLUSÕES:**

A prevalência da prática de atividade física de lazer e sua realização por ≥ 150minutos/semana aumentou no período analisado, e ambas se associaram diretamente à maior escolaridade.

## INTRODUÇÃO

A recomendação atual para prática de atividade física (AF) de intensidade moderada, de pelo menos 150 minutos semanal, da Organização Mundial da Saúde (OMS)^1^, endossada pelo Ministério da Saúde brasileiro, tem se mostrado benéfica também para gestantes, desde que não haja contraindicações^2^. Preconiza-se que esses 150 minutos sejam distribuídos ao longo da semana durante os três trimestres da gestação^2 , 3^.

Os benefícios decorrentes da prática de atividade física envolvem a proteção contra desfechos obstétricos e neonatais adversos, como ganho de peso gestacional excessivo^4^, hipertensão arterial^5^, *diabetes mellitus* gestacional^4 , 6^, parto cirúrgico^5^e macrossomia^4^. Dentre os tipos de AF durante a gestação mais frequentemente estudados na literatura, considerados seguros e benéficos, estão os exercícios aeróbicos, os de resistência e alongamento, ciclismo em cicloergômetro, natação e hidroginástica, dança, pilates^7^.

A relação entre a AF ocupacional da gestante e desfechos de saúde materno-fetal ainda precisa ser investigada, devido aos resultados divergentes e limitados dos estudos publicados^2^. Os efeitos da AF ocupacional durante a gestação nos desfechos de saúde parecem ser dependentes da carga de peso manipulado ao longo do dia, sendo que levantamento de peso superior à 20 kg, mais de 10 vezes/dia, apresentou risco aumentado de prematuridade. A recomendação do *National Institute for Occupational Safety and Health* pode ser utilizada para estimar o limite máximo de peso aceitável para uma gestante, considerando atividade ocupacional e idade gestacional^8 , 9^.

O percentual de gestantes que praticam atividade física de lazer regularmente é baixo, segundo estudos regionais brasileiros, realizados nas décadas de 2000 e de 2010^10^. Em Pelotas (RS) (2004), 12,9% das gestantes relataram praticar algum tipo de AF e houve redução da sua prática no último trimestre^11^. No interior de São Paulo, o percentual de gestantes entrevistadas em 2010 e no período de 2011–2014 que afirmou fazer AF moderada variou de 12,5%^10^a 7,6%^12^, respectivamente. Com esse quadro, nota-se que nas recomendações do Manual de Atenção ao Pré-natal de Baixo Risco, do Ministério da Saúde (2012)^13^, vigentes até a atualidade, há pouca ênfase à promoção da atividade física na gestação e nenhum destaque a seu potencial como comportamento protetor às complicações obstétricas e neonatais^13^.

Não existem dados representativos da população brasileira de gestantes e, consequentemente, e não é conhecida a tendência temporal da prática de AF na gestação, embora o monitoramento da atividade física da população em geral figure como uma das ações prioritárias da Política Nacional de Promoção da Saúde^14^. Identificou-se um estudo brasileiro que comparou dois grupos (2004 e 2015) de gestantes em Pelotas (RS). Foi observado aumento do percentual de gestantes ativas antes de engravidar, estabilidade dos percentuais de gestantes ativas nos dois primeiros trimestres gestacionais e redução da proporção ativa no terceiro trimestre^15^.

O Sistema de Vigilância de Fatores de Risco para doenças crônicas por inquérito telefônico (Vigitel) disponibiliza dados sobre atividade física. A presença de gestantes nas amostras anuais do Vigitel constitui oportunidade para conhecer o panorama da prática de AF em todas as regiões brasileiras, mesmo restringindo àquelas que residem nas capitais e no Distrito Federal (DF). Dessa forma, nossos objetivos foram descrever a tendência temporal de AF das gestantes residentes nas capitais brasileiras e no DF, entre 2007 e 2017, e verificar a associação entre fatores sociodemográficos e o alcance da recomendação de AF de lazer por gestantes a partir dos dados do Vigitel.

## MÉTODOS

### Desenho do Estudo, Fonte dos Dados e Amostra

Trata-se de estudo de tendência temporal com dados anuais do Vigitel, no período de 2007 a 2017, sendo cada inquérito com desenho transversal. O objetivo do Vigitel é monitorar de forma contínua e frequente a distribuição dos fatores de risco e proteção para doenças crônicas não transmissíveis (DCNT).

O Projeto Vigitel foi aprovado pela Comissão Nacional de Ética em Pesquisa (Conep) para Seres Humanos do Ministério da Saúde (Parecer 355590, CAAE: 16202813.2.0000.0008) e as bases de dados são públicas e estão disponíveis no website do Ministério da Saúde.

A amostra anual é obtida por sorteio de cinco mil linhas telefônicas por cidade, de forma sistemática e estratificada por CEP. A seguir, as linhas passam por segundo sorteio e são divididas em réplicas de 200 linhas, com exclusão de linhas correspondentes a empresas, que se encontram fora de serviço ou que não atendem a seis tentativas de chamadas feitas em dias e horários variados. As elegíveis passam por uma segunda etapa com sorteio de um morador adulto para responder ao questionário^16^. A adoção do método *rake* para obtenção do peso pós-estratificação permite inferência das estimativas para a população adulta residente nas capitais brasileiras e no DF, independentemente de o domicílio possuir ou não linha telefônica. Esse método foi adotado inicialmente a partir de 2012, porém foram calculados e disponibilizados também a partir de 2006^17^.

Informaram estarem grávidas 3.730 mulheres entre 2007 e 2017. Em função da amostra ser composta por gestantes, foi construído novo peso *rake* a partir da variável “peso interno”, já disponível nos bancos de dados (essa variável é construída pela multiplicação do inverso da probabilidade de adultos no domicílio pelo inverso da probabilidade de linhas telefônicas no domicílio), da faixa etária das gestantes (< 20 anos; 20–34,9 anos e ≥ 35 anos) e da capital onde residiam. Construiu-se uma variável correspondendo ao número de gestante por faixa etária e por capital. Posteriormente, o peso *rake* foi gerado a partir dessas três variáveis. A necessidade de novo peso se deve ao número pequeno de gestante na amostra de cada inquérito no período analisado e, também, em função da queda da taxa de fecundidade (de dois pontos em 2006 para aproximadamente 1,75 em 2015)^18^e de natalidade (15,8 nascimentos/mil habitantes em 2006 para 13,8 nascimentos/mil habitantes em 2016)^19^.

### Variáveis Sociodemográficas

As variáveis sociodemográficas selecionadas foram: idade (< 20 anos, 20 a 34,9 anos, ≥ 35 anos); escolaridade (zero a oito anos, nove a 11, ≥ 12 anos de estudo); trabalho (sim/não); estado civil (solteiras; viúvas/divorciadas; e casadas/união estável), macrorregião (Norte, Nordeste, Centro-Oeste, Sudeste e Sul) e cor (brancas, amarelas, pretas, pardas ou indígenas). Nas análises, optamos pelo agrupamento das gestantes em duas categorias, uma é das mulheres que se autodeclararam brancas e amarelas e a outra reunindo aquelas que se autodeclararam como pretas, pardas e indígenas^20^.

### Variáveis da Atividade Física

As variáveis disponíveis nos bancos de dados do Vigitel e analisadas neste estudo referem-se à realização de atividade física em quatro domínios: ocupacional, doméstica, deslocamento e lazer. Para os domínios AF ocupacional, doméstica e de deslocamento foram utilizadas as variáveis indicadoras previamente disponíveis em todos os bancos de dados (2007 a 2017) e cuja configuração foi “não/sim”.

A categoria *sim* para AF ocupacional corresponde à gestante ter trabalhado nos últimos três meses e ter relatado andar bastante e/ou carregar peso no trabalho. A categoria *sim* para AF no deslocamento, corresponde à gestante ter caminhado 30 minutos ou mais entre o trabalho e/ou curso/escola. A categoria *sim* para AF doméstica corresponde realizar a faxina sozinha e/ou fazer sozinha a parte pesada da faxina.

A AF no lazer foi configurada pelos autores a partir da variável “Nos últimos três meses, a sra. praticou algum tipo de exercício físico ou esporte?” (sim/não). A variável tipo de AF de lazer só estava disponível para as gestantes que responderam praticar algum tipo de exercício físico ou esporte nos últimos três meses e tê-lo feito mais de uma vez por semana. As diversas modalidades de AF foram agrupadas em quatro categorias, segundo a classificação da intensidade do gasto energético em múltiplos de equivalentes metabólicos (MET)^21^: caminhada na rua e na esteira; dança, ginástica, hidroginástica, musculação, natação; bicicleta, futebol, lutas marciais, voleibol, tênis; corrida na rua e em esteira.

A variável AF de lazer ≥ 150min por semana (AF ≥ 150min/semana) foi configurada pelos autores a partir da combinação de duas variáveis: frequência semanal da AF (número de dias/semana) e a duração da AF (minutos/seção). Foram incluídas na categoria “sim” as gestantes que relataram praticar algum exercício físico/esporte nos últimos três meses com frequência acima de uma vez por semana e cuja soma da frequência semanal e duração resultasse em ≥ 150min/semana. As gestantes que não faziam atividade física ou que faziam somente uma vez na semana foram classificadas como “não” para essa variável. Assim, foi possível estimar a prevalência da prática de atividade física conforme recomendação da OMS^1^.

### Análise dos Dados

Os dados foram descritos em prevalência por ano do inquérito e a análise da tendência temporal foi realizada por regressão linear ponderada pela variância. A taxa média de aumento ou redução (expressos em pontos percentuais – p.p.) da prevalência entre 2007 e 2017 corresponde ao valor do coeficiente de inclinação da regressão com o respectivo p-valor de tendência (nível de significância menor de 5%).

A prevalência da prática de atividade física no lazer (sim/não) e da prática de AF ≥ 150min/sem (sim/não), segundo a escolaridade, foram ilustradas em um gráfico definido como *equiplot* .

A associação entre a escolaridade da gestante e a realização da atividade física de lazer e AF ≥ 150min/sem foi estimada por análise de regressão de Poisson, ajustada por faixa etária, cor, macrorregião geográfica e estado civil. Foram ajustados dois modelos para cada desfecho: a) modelo 1 com a inclusão de todos os anos dos inquéritos (2007 a 2017) e b) modelo 2 com divisão da amostra em três períodos (2007 a 2009; 2010 a 2014 e 2015 a 2017). A opção por analisar os modelos nesses três períodos deve-se à redução da amostra ao longo dos anos, levando ao aumento dos intervalos de confiança nas análises e redução da precisão das estimativas. Todas as análises foram realizadas no programa Stata SE 13.0^®^.

## RESULTADOS

A amostra total coletada no período de 2007 a 2017 foi de 3.730 gestantes (variação de 2% a 1,38% da amostra total de mulheres entrevistadas em cada inquérito), com tamanhos semelhantes e maiores no período de 2007 (n = 462 gestantes) a 2011 (n = 396 gestantes) e redução a partir de 2012 (n = 317 em 2012 a n = 188 em 2017).

Observamos aumento do percentual de gestantes com ≥ 35 anos no período analisado, porém a maioria das gestantes tinha entre 20 e 34,9 anos ao longo do período. Houve aumento significativo da prevalência de gestantes com ≥ 12 anos, sendo que a maioria em 2017 foi classificada nessa faixa de escolaridade, em oposição à 2007, quando a categoria de nove a 11 anos de estudo foi mais prevalente. A prevalência de gestantes que relataram estar trabalhando aumentou de 58,6% para 67,8% entre 2007 e 2017. As maiores prevalências de gestantes no período de 2007 a 2017 foram identificadas nas regiões Norte e Nordeste, com aumento, no mesmo período, de gestantes residentes na região Nordeste e redução no Centro-Oeste e Sul. A maioria das gestantes relatou estar casada ou em união estável. Houve maior prevalência, em todo o período analisado, das gestantes que se autodeclararam pretas, pardas e indígenas (59% em 2007 e 61,3% em 2017) ( Tabela 1 ).

**Tabela 1:** Características sociodemográficas das gestantes brasileiras residentes nas capitais brasileiras e no Distrito Federal, segundo ano do inquérito. Vigitel, 2007–2017.

Anos do inquérito												
Variáveis	2007	2008	2009	2010	2011	2012	2013	2014	2015	2016	2017	Incremento médio/ano (em p.p.) (p)
Faixa etária (anos)												
< 20	5,7	4,6	4,9	7,5	8,8	5,9	5,4	3,9	2,6	7,2	6,2	-0,12 (0,288)
20 a 35	74,7	73,7	76,3	71,9	75,4	74,4	69,4	68,7	69,1	65,7	59,4	-1,09 (< 0,001)
≥ 35	19,6	21,7	18,8	20,6	15,8	19,7	25,2	27,4	28,3	27,1	34,4	1,11 (< 0,001)
Escolaridade (anos)												
0 a 8	17,2	14,9	11,3	18,4	17,3	12,6	11,6	13,1	8,9	7,5	8,9	-0,79 (< 0,001)
9 a 11	49,0	46,2	50,9	47,8	46,7	48,7	46,6	45,7	49,7	39,0	40,8	-0,59 (0,040)
≥ 12	33,8	38,9	37,8	33,8	36,0	38,7	41,8	41,2	41,4	53,5	50,3	1,37 (< 0,001)
Trabalho												0,75 (0,007)
Não	41,4	36,6	37,1	37,2	40,7	38,8	39,0	37,6	27,1	35,0	32,2	
Sim	58,6	63,4	62,9	62,8	59,3	61,2	61,0	62,4	72,9	65,0	67,8	
Macrorregião												
Norte	31,7	31,3	38,4	36,0	34,4	31,5	34,8	27,9	30,8	31,4	31,6	-0,29 (0,295)
Nordeste	26,3	33,6	26,9	26,6	32,0	33,0	29,4	36,6	40,9	30,2	39,3	0,97 (< 0,001)
Centro-Oeste	19,2	13,0	15,0	15,2	16,3	13,5	13,4	13,4	10,3	15,8	11,0	-0,48 (0,015)
Sudeste	12,9	14,2	11,8	11,7	10,2	12,4	12,0	14,9	12,1	14,5	14,3	0,08 (0,705)
Sul	9,9	7,9	7,9	10,5	7,1	9,6	10,4	7,2	5,9	8,1	3,8	-0,41 (0,003)
Estado civil												0,49 (0,075)
Solteira, viúva, divorciada	23,5	33,9	32,6	31,7	35,7	28,3	34,5	28,8	27,9	37,2	34,9	
Casada, união estável	76,5	66,1	67,4	68,3	64,3	71,7	65,5	71,2	72,1	62,8	65,1	
Cor												-0,08 (0,782)
Branca/amarelas	41,0	35,7	41,7	39,4	40,8	44,7	40,6	39,4	36,6	43,5	38,7	
Pretas, pardas, indígenas	59,0	64,3	58,3	60,6	59,2	55,3	59,4	60,6	63,4	56,5	61,3	

p.p.: pontos percentuais.

A prevalência de gestantes que relataram prática de alguma atividade física no trabalho (AF ocupacional) foi estável ao longo do período, em torno de 32%. Houve discreto aumento da AF no deslocamento entre domicílio e trabalho/estudo ao longo do período (+0,34 p.p./ano) e redução expressiva da atividade física realizada no trabalho doméstico (-1,65 p.p./ano). Observamos aumento da AF classificada como de lazer (+1,37 p.p./ano), assim como do percentual de gestantes que relataram praticar AF ≥ 150min/sem (+2,33 p.p./ano) ( Tabela 2 ). Verificamos que, em 2007, a AF doméstica foi a mais prevalente, com dados semelhantes aos das AF no lazer e ocupacional. Entretanto, em 2017, as prevalências de AF doméstica e de AF lazer foram virtualmente iguais e superiores às AF ocupacional e de deslocamento.

**Tabela 2:** Tendência da prática de AF nos quatro domínios (lazer, trabalho, deslocamento e doméstico) e prática de AF segundo recomendação OMS (150 min/semana) de gestantes brasileiras residentes nas capitais brasileiras e Distrito Federal, segundo ano do inquérito. Vigitel, 2007–2017.

Anos do inquérito												
Variáveis	2007	2008	2009	2010	2011	2012	2013	2014	2015	2016	2017	Incremento médio/ano (em p.p.) (p)
Trabalho												0,15 (0,593)
Não	68,3	62,9	65,7	62,3	65,5	64,7	66,6	64,2	59,3	64,3	68,4	
Sim	31,7	37,1	34,3	37,7	34,5	35,3	33,4	35,8	40,7	35,7	31,6	
Deslocamento												0,34 (0,038)
Não	94,9	89,3	88,6	84,2	85,8	88,7	89,6	88,1	90,1	90,7	91,3	
Sim	5,1	10,7	11,4	15,8	14,2	11,3	10,4	11,9	9,9	9,3	8,7	
Atividades domésticas												-1,65 (< 0,001)
Não	36,1	52,5	48,9	48,3	49,6	51,2	54,2	51,3	56,9	55,4	61,6	
Sim	63,9	47,5	51,1	51,7	50,4	48,8	45,8	48,7	43,1	44,6	38,4	
Lazer												1,37 (< 0,001)
Não	70,7	75,8	71,4	76,9	77,6	69,5	66,0	66,8	64,5	56,6	62,4	
Sim	29,3	24,2	28,6	23,1	22,4	30,5	34,0	33,2	35,5	43,4	37,6	
AF (> 150min/sem)												2,33 (< 0,001)
Não	97,7	87,1	84,2	87,7	87,3	82,5	78,8	82,1	78,3	73,7	79,4	
Sim	2,3	12,9	15,8	12,3	12,7	17,5	21,2	17,9	21,7	26,3	20,6	

AF: atividade física; p.p.: pontos percentuais.

Os tipos de AF de lazer praticados pelas gestantes foram semelhantes em todos os anos, dessa forma, optamos por descrever os resultados de todo o período. As atividades mais prevalentes foram caminhada na rua e na esteira (57,8%); seguidas da categoria “dança, ginástica, hidroginástica, musculação e natação” (32,7%); com menores prevalências das categorias “bicicleta, futebol, lutas marciais, tênis e voleibol” (7,2%) e “corrida na rua e na esteira” (2,3%).

As prevalências de AF de lazer e da AF ≥ 150min/sem foram maiores entre as gestantes com ≥ 12 anos de escolaridade no período analisado. Para essas duas variáveis, a prevalência foi superior a 30% entre as gestantes com maior escolaridade. Entre as gestantes com menos de oito anos de escolaridade, a prevalência de AF ≥ 150min/sem foi inferior a 10% em quase todos os anos do período analisado ( Figura ).

**Figura:** Equiplot da prática da atividade física (AF) de lazer e da prática de AF por 150 min/semana ou mais segundo escolaridade em gestantes residentes nas capitais brasileiras e no Distrito Federal. Vigitel, 2007–2017.

Houve aumento da prática das atividades físicas de lazer e da AF ≥ 150min/sem no período de 2007 a 2017 ( Tabela 3 ). Verificamos que a prática de AF no lazer foi 2,22 vezes mais prevalente entre as gestantes com ≥ 12 anos de escolaridade em comparação àquelas com menos de oito anos (modelo 1). Esse padrão se repetiu nos três períodos analisados (modelo 2). As gestantes que se autodeclararam pretas/pardas/indígenas relataram menor prevalência de atividade física no modelo 1 e no período de 2015 a 2017 (modelo 2). Não observamos diferença de prevalência segundo região geográfica e idade (modelos 1 e 2).

**Tabela 3:** Associação entre escolaridade e prática de AF de lazer nos últimos 3 meses e AF por 150min/semana ou mais em gestantes residentes nas capitais brasileiras e no Distrito Federal, no período de 10 anos (modelo 1) e intervalos anuais (modelo 2). Vigitel, 2007–2017.

	AF no lazer				AF > 150min/sem			
	Modelo 1	Modelo 2			Modelo 1	Modelo 2		
	2007–2017 RP (IC95%)	2007–2009 RP (IC95%)	2010–2014 RP (IC95%)	2015–2017 RP (IC95%)	2007–2017 RP (IC95%)	2007–2009 RP (IC95%)	2010–2014 RP (IC95%)	2015–2017 RP (IC95%)
Ano	1,04 (1,02–1,06)				1,11 (1,08–1,14)			
Escolaridade (anos)								
0 a 8	1,00 (ref)	1,00 (ref)	1,00 (ref)	1,00 (ref)	1,00 (ref)	1,00 (ref)	1,00 (ref)	1,00 (ref)
9 a 11	1,66 (1,30–2,13)	1,96 (1,30–2,95)	1,53 (1,09–2,16)	1,60 (0,79–3,22)	1,93 (1,33–2,80)	3,98 (1,82–8,73)	1,55 (0,98–2,46)	2,15 (0,86–5,37)
≥ 12	2,22 (1,73–2,84)	2,41 (1,60–3,64)	2,12 (1,51–2,98)	2,29 (1,15–4,58)	2,36 (1,63–3,42)	4,56 (2,08–10,0)	1,95 (1,22–3,09)	2,84 (1,16–6,99)
Cor								
Branca/amarela	1,00 (ref)	1,00 (ref)	1,00 (ref)	1,00 (ref)	1,00 (ref)	1,00 (ref)	1,00 (ref)	1,00 (ref)
Pretas, pardas, indígenas	0,87 (0,78–0,97)	0,86 (0,70–1,05)	1,00 (0,84–1,19)	0,64 (0,52–0,79)	0,91 (0,76–1,08)	0,81 (0,57–1,16)	1,03 (0,80–1,32)	0,79 (0,56–1,07)
Macrorregião								
Norte	1,00 (ref)	1,00 (ref)	1,00 (ref)	1,00 (ref)	1,00 (ref)	1,00 (ref)	1,00 (ref)	1,00 (ref)
Nordeste	0,85 (0,74–0,98)	0,82 (0,64–1,04)	0,81 (0,64–1,02)	0,97 (0,75–1,24)	0,86 (0,69–1,08)	0,66 (0,41–1,06)	0,93 (0,66–1,32)	0,97 (0,66–1,42)
Centro-Oeste	1,15 (0,98–1,34)	1,02 (0,77–1,34)	1,23 (0,97–1,56)	1,24 (0,93–1,65)	1,34 (1,05–1,70)	0,93 (0,57–1,53)	1,54 (1,08–2,19)	1,52 (0,99–2,32)
Sudeste	1,03 (0,86–1,23)	0,86 (0,62–1,20)	1,28 (1,00–1,65)	0,86 (0,61–1,21)	1,18 (0,90–1,53)	0,82 (0,46–1,47)	1,53 (1,05–2,23)	1,08 (0,66–1,75)
Sul	1,19 (0,99–1,44)	1,19 (0,87–1,61)	1,36 (1,02–1,80)	0,86 (0,57–1,31)	1,43 (1,06–1,92)	1,25 (0,73–2,15)	1,75 (1,14–2,67)	1,01 (0,55–1,85)
Idade (anos)								
< 20	1,00 (ref)	1,00 (ref)	1,00 (ref)	1,00 (ref)	1,00 (ref)	1,00 (ref)	1,00 (ref)	1,00 (ref)
20 a 34	1,15 (0,88–1,50)	0,95 (0,62–1,47)	1,51 (0,96–2,38)	0,82 (0,51–1,32)	1,78 (1,10–2,88)	1,55 (0,60–3,99)	1,72 (0,88–3,36)	2,15 (0,80–5,82)
≥ 35	1,28 (0,97–1,69)	1,05 (0,66–1,67)	1,83 (1,14–2,94)	0,82 (0,50–1,34)	2,17 (1,32–3,58)	2,16 (0,81–5,78)	2,40 (1,19–4,80)	2,12 (0,77–5,80)

AF: atividade física; RP: razão de prevalência; IC95%: intervalo de confiança de 95%.

A prática da AF ≥ 150min/sem foi 2,36 vezes mais prevalente entre as gestantes com ≥ 12 anos de escolaridade em comparação àquelas com menos de oito anos (modelo 1), sendo que os valores da razão de prevalência foram mais elevados especialmente no período de 2007 a 2009. Observamos também maior prevalência de AF nas gestantes com idade > 20 anos (modelo 1) e aquelas que residiam nas regiões Centro-Oeste e Sul (modelo 1, modelo 2 – 2010 a 2014) ( Tabela 3 ).

## DISCUSSÃO

Neste estudo, observamos redução expressiva do número de gestantes que relataram fazer AF domésticas, aumento de gestantes que faziam AF no lazer e que alcançaram 150min/semana no período analisado. Entretanto, observamos também que a prática de AF no lazer, bem como a realização da AF ≥ 150min/sem são superiores entre as gestantes com maior escolaridade, apontando importante desigualdade social entre as gestantes brasileiras.

A AF ocupacional se manteve estável ao longo da década analisada e a AF no deslocamento apresentou pequeno aumento. Já o percentual de gestantes que relatou fazer atividades físicas domésticas reduziu pela metade. O aumento mais expressivo no período foi no domínio AF no lazer, componente do conjunto de AF mais modificável pelas ações de promoção de saúde.

A relação entre atividades físicas domésticas e ocupacionais com o risco de prematuridade, baixo peso ao nascer e restrição de crescimento intrauterino dominou as pesquisas das décadas de oitenta e noventa, em especial em países de baixa renda, onde tais desfechos eram e ainda são um problema de saúde pública. Os resultados daquela época indicavam que o excesso de horas em pé, subindo e descendo escadas e carregando peso, cotidianamente, reduz o crescimento do conceito e a idade gestacional no parto^22^. Esses resultados ainda continuam aceitos e, portanto, a redução das gestantes ativas nesses domínios pode ser vista como positiva^2^.

É possível que os efeitos dos diferentes domínios de atividades físicas variem conforme o contexto de vida das gestantes, como apontaram dois estudos recentes^23 , 24^. Em dois estudos de coortes com gestantes francesas, altos níveis de AF domésticas e de atividades sedentárias no terceiro trimestre gestacional aumentaram as chances de depressão pós-parto, enquanto exercícios e atividades esportivas não se associaram a esse desfecho^23^. Em coorte vietnamita, maiores níveis de atividades domésticas e ocupacionais associaram-se com menor ganho de peso gestacional. No contexto local, esse resultado foi considerado favorável à prevenção do diabetes gestacional, problema frequente nessa população, mas negativo para a ocorrência de recém-nascidos com baixo peso^24^.

Por outro lado, a literatura contém evidências sólidas sobre os benefícios da prática regular de AF de lazer e exercícios durante a gestação^2^, reforçando os aspectos positivos das tendências observadas. Contudo, o aumento médio foi maior no início do período (2007–2009) (4,5 p.p./ano) e menor no final (0,55 p.p./ano) 2015 e 2017.

Não há meta nacional ou internacional para alcance de determinado percentual de gestantes realizando AF de lazer ≥ 150 minutos/semanais. É possível estimar que o ritmo de aumento observado no período 2007–2017 é baixo se levarmos em conta que, mantendo-se esse padrão, levará mais de 40 anos para que metade das gestantes brasileiras alcance a recomendação. Esse tempo poderá ser ainda maior se o ritmo de aumento for o observado nos anos mais recentes (2014 a 2017). Esse quadro indica o tamanho do desafio a ser enfrentado no Brasil para promover e apoiar as mulheres a serem fisicamente ativas durante a gestação. Essa situação ressalta a importância de, além do monitoramento, identificar os determinantes da prática de atividade física no lazer de mulheres durante a gestação, visando fornecer evidências para a condução de intervenções.

As barreiras à realização da AF de lazer por gestantes têm sido objeto de investigações^24 , 25^. Uma revisão da literatura com 26 estudos qualitativos e quantitativos sobre barreiras para a realização de atividade física na gestação identificou em todos eles fatores intrapessoais de impedimento da prática, incluindo cansaço e desconfortos físicos, além do excesso de atividades e responsabilidades com trabalho e o cuidado com a família. Também foram frequentes fatores interpessoais, com destaque para a falta de conhecimento e orientação sobre a importância e segurança da atividade física nesse período^26^. Essas barreiras poderiam ser removidas pelos profissionais responsáveis pelo pré-natal. Barreiras relacionadas ao ambiente organizacional, político e ao clima (muito frio ou muito quente, chuvoso) também foram identificadas pelos estudos, em especial a insegurança nas ruas, a falta de espaços públicos para a realização de atividade física e o alto custo das academias^27^.

Assim, para superar as barreiras, cabem ações nos serviços de saúde e ações intersetoriais que vão além do aconselhamento realizado por profissionais de saúde. Remover tais barreiras implica também novas perspectivas para planejamento urbano, ambientes de trabalho, divisão do trabalho doméstico, entre outras esferas da vida humana que precisam se tornar mais facilitadoras do desempenho da atividade física por todos, inclusive as gestantes. Políticas públicas em todas essas esferas são necessárias, principalmente, para apoiar as gestantes autodeclaradas pretas, pardas e indígenas e aquelas com menor escolaridade.

O aumento da escolaridade de gestantes tende a ser favorável ao alcance da recomendação de AF no lazer, conforme estudos prévios também verificaram^12 , 28^. A associação positiva entre maior escolaridade e AF lazer pode ser explicada pela condição socioeconômica mais favorável, o que proporciona trabalharem em atividades que exigem menos esforço físico, obterem ajuda com os serviços domésticos e com o cuidado de crianças, ou seja, ter mais tempo e disposição de se exercitarem no tempo de lazer^31^. Além disso, gestantes com maior nível educacional podem ter mais acesso a informações técnicas sobre atividade física durante a gravidez, ter acesso facilitado a locais mais seguros e com melhor infraestrutura para prática de AF lazer. As diferenças de AF lazer entre gestantes brancas/amarelas e pretas/pardas/indígenas, apontam para a desigualdade relacionada ao racismo institucional, independente da escolaridade, na prática da AF no lazer.

Destacamos como pontos positivos do nosso estudo a produção de dados inéditos sobre o perfil da atividade física de gestantes das capitais brasileiras, ampliando assim o conhecimento desse perfil para as cinco macrorregiões. Até o presente momento, os estudos prévios sobre atividade física de gestantes eram provenientes de dados regionais^10 , 11 , 15^. Outro ponto positivo é a utilização dos dados de um sistema de monitoramento (Vigitel) disponível com as mesmas questões em todos os anos dos inquéritos, o que permitiu comparabilidade entre os anos analisados.

Identificamos como principal limitação a ausência de algumas variáveis específicas e relevantes ao realizarmos estudos com gestantes, especialmente a idade gestacional. Essa variável nos permitiria identificar diferenças relativas à prática de atividade física por trimestres gestacionais e estado nutricional. O Vigitel não tem como foco as gestantes e sim a população adulta residente nas capitais brasileiras e no DF. Ainda assim, sugerimos que a idade gestacional seja indagada nos inquéritos futuros, uma vez que o Vigitel é o único inquérito de base populacional disponível no Brasil atualmente que permite analisar atividade física de gestantes. Essa simples informação permitirá avanços no monitoramento de comportamentos de risco e de proteção também para gestantes e o delineamento de intervenções específicas a cada fase da gestação.

Podemos destacar também que a amostra de gestantes deste estudo não é representativa da população de gestantes que residem nas capitais brasileiras, uma vez que a população a ser representada são indivíduos com 18 anos ou mais. Uma das possíveis explicações para a redução do tamanho da amostra é queda da fertilidade ao longo dessa década, especialmente nas capitais.

Outra limitação a ser considerada é o autorrelato da atividade física em comparação às medidas diretas da AF, realizadas por pedômetros e/ou acelerômetros. No entanto, são comparáveis os estudos de tendência e monitoramento que utilizam as mesmas formas de coleta das informações. Ressalvamos que ao classificar as gestantes quanto à frequência e duração semanal da AF de lazer consideramos todas as modalidades como moderadas, já que estudo prévio^12^indica baixa frequência de gestantes que realizam atividades de intensidade vigorosa.

Em síntese, verificamos tendência de aumento da atividade física entre as gestantes nos domínios lazer e no deslocamento e redução expressiva das atividades domésticas. Adicionalmente, houve tendência de aumento daquelas que alcançaram a recomendação de ≥ 150min/semana. Entretanto, esse aumento foi decrescente no período e desigual entre as gestantes, de acordo com a escolaridade, sendo superior entre as gestantes com 12 anos de estudo ou mais e entre aquelas que se autodeclararam brancas/amarelas em todo o período analisado.
